# Suppression of endogenous lipogenesis induces reversion of the malignant phenotype and normalized differentiation in breast cancer

**DOI:** 10.18632/oncotarget.9463

**Published:** 2016-05-18

**Authors:** Anatilde M. Gonzalez-Guerrico, Ingrid Espinoza, Barbara Schroeder, Cheol Hong Park, KVP Chandra Mohan, Ashwani Khurana, Bruna Corominas-Faja, Elisabet Cuyàs, Tomás Alarcón, Celina Kleer, Javier A. Menendez, Ruth Lupu

**Affiliations:** ^1^ Department of Laboratory Medicine and Pathology, Division of Experimental Pathology, Mayo Clinic, Rochester, MN, USA; ^2^ Cancer Institute, University of Mississippi Medical Center, Jackson, MS, USA; ^3^ Department of Biochemistry, University of Mississippi Medical Center, Jackson, MS, USA; ^4^ ProCURE (Program Against Cancer Therapeutic Resistance), Metabolism and Cancer Group, Catalan Institute of Oncology, Girona, Catalonia, Spain; ^5^ Molecular Oncology Group, Girona Biomedical Research Institute (IDIBGI), Girona, Spain; ^6^ Computational and Mathematical Biology Research Group, Centre de Recerca Matemàtica (CRM), Barcelona, Spain; ^7^ Departament de Matemàtiques, Universitat Autònoma de Barcelona (UAB), Barcelona, Spain; ^8^ ICREA (Institució Catalana d'Estudis i Recerca Avançats), Barcelona, Spain; ^9^ Barcelona Graduate School of Mathematics (BGSMath), Barcelona, Spain; ^10^ Department of Pathology, University of Michigan Medical School, Ann Arbor, MI, USA; ^11^ Mayo Clinic Cancer Center, Rochester, MN, USA

**Keywords:** fatty acid synthase, lipogenesis, cancer, tumor reversion, phenotype

## Abstract

The correction of specific signaling defects can reverse the oncogenic phenotype of tumor cells by acting in a dominant manner over the cancer genome. Unfortunately, there have been very few successful attempts at identifying the primary cues that could redirect malignant tissues to a normal phenotype. Here we show that suppression of the lipogenic enzyme fatty acid synthase (FASN) leads to stable reversion of the malignant phenotype and normalizes differentiation in a model of breast cancer (BC) progression. FASN knockdown dramatically reduced tumorigenicity of BC cells and restored tissue architecture, which was reminiscent of normal ductal-like structures in the mammary gland. Loss of FASN signaling was sufficient to direct tumors to a reversed phenotype that was near normal when considering the development of polarized growth-arrested acinar-like structure similar to those formed by nonmalignant breast cells in a 3D reconstituted basement membrane *in vitro*. This process, *in vivo*, resulted in a low proliferation index, mesenchymal-epithelial transition, and shut-off of the angiogenic switch in FASN-depleted BC cells orthotopically implanted into mammary fat pads. The role of FASN as a negative regulator of correct breast tissue architecture and terminal epithelial cell differentiation was dominant over the malignant phenotype of tumor cells possessing multiple cancer-driving genetic lesions as it remained stable during the course of serial *in vivo* passage of orthotopic tumor-derived cells. Transient knockdown of FASN suppressed hallmark structural and cytosolic/secretive proteins (vimentin, N-cadherin, fibronectin) in a model of EMT-induced cancer stem cells (CSC). Indirect pharmacological inhibition of FASN promoted a phenotypic switch from basal- to luminal-like tumorsphere architectures with reduced intrasphere heterogeneity. The fact that sole correction of exacerbated lipogenesis can stably reprogram cancer cells back to normal-like tissue architectures might open a new avenue to chronically restrain BC progression by using FASN-based differentiation therapies.

## INTRODUCTION

The aberrant oncogenic phenotype of tumor cells can revert to a normalized, non-malignant state without necessarily correcting the cancer-driving genomic abnormalities [1–6]. Even when genetic abnormalities elicit defects in the stem cell-like population, driving tumor maintenance and metastasis, these life-threatening cells do not always lose their ability to undergo differentiation [7]. Studies with myeloid leukemia cells have shown that the epigenetic reprogramming of malignant cells, by inducing differentiation, can efficiently bypass cancer-driving genetic abnormalities [8–11]. The therapeutic paradigm of differentiation therapy as a valid approach for “phenotypic reversion” in the treatment of cancer patients is exemplified by the successful use of all-trans retinoic acid (ATRA) as the regimen of choice for treatment of acute promyelocytic leukemia (APL). Nevertheless, beyond the occurrence of complete remissions in patients with APL treated with ATRA, there have been very few successful attempts at identifying the signaling defects that, once corrected, might successfully favor the process of halting malignancy by overriding the genetic abnormalities in tumor cells. However, interest and application of differentiation-based therapy as a viable treatment modality for the clinical management of solid malignancies have lagged mostly due to deficiencies in our understanding of differentiation pathways in solid malignancies. It therefore remains an urgent task of cancer researchers to identify the primary signaling transduction pathways whose normalization would lead to reversions of the malignant phenotype for the most common types of human carcinomas.

Cancer is beginning to be understood as a disease of reprogramming that involves the progressive resetting of the metabolic infrastructure and metabolite levels concomitantly with changes in cellular differentiation [12–18]. Modulation of metabolism and associated signaling is increasingly postulated to be vital in the determination of cell identity during oncogenesis, i.e., metabolism *per se* can dictate cancer cell fate decisions and differentiation outcomes. Because activation of fatty acid synthase (FASN), a key lipogenic enzyme catalyzing the terminal steps of *de novo* fatty acid (FA) biogenesis, is an early and near universal hallmark of most human carcinomas and their precursor lesions [19–25], we hypothesized that the correction of exacerbated endogenous lipogenesis might be sufficient to stably revert the malignant phenotype. We provide evidence that the sole correction of exaggerated lipogenesis leads to a stable phenotypic reversion and normalized differentiation of malignant tissue by acting in a dominant manner over the unstable cancer genome in a model of breast cancer (BC) progression. The discovery of FASN signaling as a hitherto unrecognized organizer of breast tissue architecture can provide new therapeutic avenues aimed to chronically restrain the life-threatening potential of invasive carcinomas by using FASN-based differentiation therapies.

## RESULTS

### FASN expression status correlates with the malignant phenotype during BC progression

**Figure 1:**
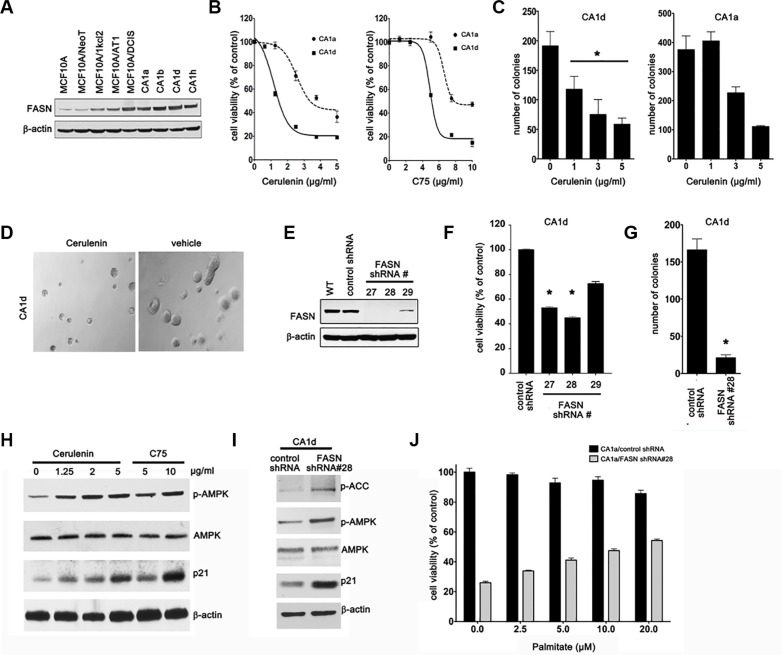
FASN expression correlates with malignant progression of MCF10CA cells (**A**) Western blot analysis of FASN protein in cells from various tumor stages. NeoT: neoplasmic, kcl2: atypical hyperplasia, AT1: hyperplasia, DCIS: ductal carcinoma *in situ*, CA: metastatic/invasive cancer. (**B**) Inhibition of FASN reduces cell viability. CA1d and CA1a cells were treated with cerulenin or C75 and cell viability was assessed by MTT reduction. Dose–response curves were plotted as percentages of the control cells' absorbance (= 100%). Results from one representative experiment are presented as mean ± SD; *p* ≤ 0.02 for cerulenin and *p* ≤ 0.05 for C75, respectively. (**C**) FASN inhibition impairs anchorage-independent growth. CA1d and CA1a cells were grown in soft agar in the presence of increasing concentrations of cerulenin for 21 d. Colony numbers from one representative image are shown as mean ± SD; *p < 0.05. (**D**) Representative images from a soft agar growth assay as described in C. (**E**) FASN knockdown efficiency in stably-transduced CA1d cells; WT = wild type. (**F**) Depletion of FASN decreases cell viability. Control or FASN-depleted cells were assessed by MTT reduction 72 h post transfection and the results from one representative experiment are presented as mean ± SD; *p ≤ 0.05. (**G**) FASN depletion inhibits anchorage-independent growth. FASN-depleted or control CA1d cells were grown in soft agar and colony numbers from one representative experiment are plotted as mean ± SD, *p < 0.01. (**H**) Inhibition of FASN increases the levels of energy stress markers. CA1d cells were treated with either cerulenin or C75 and cell lysates were subjected to western blotting. Note a substantial increase in p-AMPK and p21 upon FASN inhibition. (**I**) Depletion of FASN increases the levels of energy stress markers. Control and FASN-depleted CA1d cells were lysed and subjected to western blotting. (**J**) Palmitate increases cell viability of FASN-depleted cells. CA1d/vector control or FASN-depleted cells were exposed to increasing concentrations of palmitate-BSA in 0.5% HS containing medium and cell viability was assessed by MTT reduction 72 h after treatment. Data are presented as mean ± SD from two independent experiments.

We took advantage of a powerful model of multiple cancer cell lines derived from the spontaneously immortalized mammary epithelial MCF10A cell line. The MCF10A progression series, including MCF10A untransformed cells, MCF10AneoT and MCF10AT non-malignant cells, MCF10DCIS.com ductal carcinoma *in situ* cells and MCF10Ca1a, Ca1d and Ca1h malignant cell lines, covers the entire spectrum of BC progression, ranging from non-transformed breast epithelial cells to metastatic BC cells [26–28]. Strikingly, the increase in the tumorigenic and invasive potential of MCF10A-derived BC series positively correlated with an incremental increase in the expression levels of FASN protein (Figure 1A), suggesting that up-regulation of FASN-dependent endogenous lipogenesis accompanies aggressiveness in BC phenotypes. The highly aggressive and metastatic cell lines CA1a and CA1d (short for MCF10A-CA1a and MCF10A-CA1d, respectively) showed the greatest expression of FASN protein (Figure 1A). These *in vitro* findings are in accordance with previous clinical studies demonstrating that FASN expression increases as BC progresses towards more advanced stages.

We tested if FASN acts as a metabolic oncogene capable of inducing transformation of breast epithelial cells. We first examined the sensitivity of immortalized but otherwise normal MCF10A cells to FASN inhibitors [29]. Neither cerulenin nor C75 significantly affected viability of MCF10A cells (Figure S1A, S1B). MCF10A cells engineered to stably overexpress FASN (MCF10A/FASN cells, Figure S1C) demonstrated a significantly augmented proliferation capacity (Figure S1E) and, more importantly, gained the capacity for anchorage-independent growth in soft agar (Figure S1F). Additionally, MCF10A/FASN cells were remarkably sensitive to the FASN inhibitor C75 compared with MCF10A parental cells (Figure S1D). Collectively, these findings, strongly support the notion that overexpression of FASN contributes to the development of the malignant phenotype during BC progression.

To further evaluate the involvement of FASN during BC progression, we monitored the impact of FASN pharmacological inhibition on cell viability of the most malignant and aggressive cell lines from the MCF10A series. In contrast to MCF10A parental cells, the aggressive derivatives obtained from xenograft-passaging in nude mice [30], CA1a and CA1d, were exquisitely sensitive to low concentrations of FASN inhibitors as demonstrated by a decrease in cell viability (Figure 1B) and a reduction in anchorage-independent growth (Figure 1C, 1D). Cells depleted for FASN protein using various lentiviral vectors encoding shRNAs specific for the FASN gene (Figure 1E) largely recapitulated the growth phenotype of CA1d cells treated with pharmacological FASN inhibitors (Figure 1F, 1G). Moreover, stable shRNA-mediated depletion of FASN reproduced the previously described ability of pharmacological FASN inhibitors to significantly perturb the cellular energy status, leading to the AMPK-related phosphorylation and inactivation of acetyl coenzyme A carboxylase (ACC) [31] (Figure 1H, 1I).

To question whether FASN inhibition was responsible for the growth phenotype of FASN-depleted aggressive BC cells, we reevaluated their cell proliferation capacity in the presence of palmitate, the product of FASN action. Exogenously added palmitate noticeably restored cell viability of FASN-depleted CA1d cells in a dose-dependent manner (Figure 1J). Furthermore, pharmacological blockade of FASN activity failed to further inhibit the growth of FASN-depleted CA1d cells (Figure S2A, S2B), thus confirming that FASN knockdown efficiently normalized the aberrant proliferation driven by overexpression of FASN in CA1d parental cells.

### Suppression of FASN-driven endogenous lipogenesis restores a non-malignant phenotype to aggressive BC cells *in vitro*

Profound morphological differences were observed between FASN-depleted and FASN-overexpressing CA1d parental cells after culture for 10 days in a 3D reconstituted basement membrane [32–34] (Figure S3A). Accordingly, CA1d control cells formed large, loosely organized colonies similar to those formed by primary tumor cells [33]. The failure of CA1d cells to undergo normal morphogenesis in basement membrane was also demonstrated by their compromised degree of cell-cell adhesion as shown by the absence of lateral staining of the epithelial marker E-cadherin (Figure S3A, *top*) and by an increased cytoplasmic localization of E-cadherin (cell fractionation studies; not shown). Interestingly, upon closer inspection, FASN-depleted CA1d cells appeared to have truly reverted to a “non-malignant” phenotype (Figure S3A, *bottom*). Thus, after 10 days in Matrigel, CA1D/FASN shRNA#28 cells underwent morphogenesis and formed small, highly organized acini reminiscent of those formed by cells from reduction mammoplasty [33]. Nevertheless, CA1d and CA1D/FASN shRNA#28 cells expressed essentially the same levels of E-cadherin (Figure S3B), suggesting that normalization of the malignant phenotype in FASN-depleted CA1d cells was associated with a correct re-assembly of adherent junctions rather than with significant up-regulation of cell adhesion proteins. The mesenchymal markers vimentin and N-cadherin, however, were decreased in FASN-depleted CA1d cells when compared with CA1d parental cells (Figure S3B).

### FASN inhibition promotes normalized differentiation of metastatic BC cells *in vivo*

**Figure 2:**
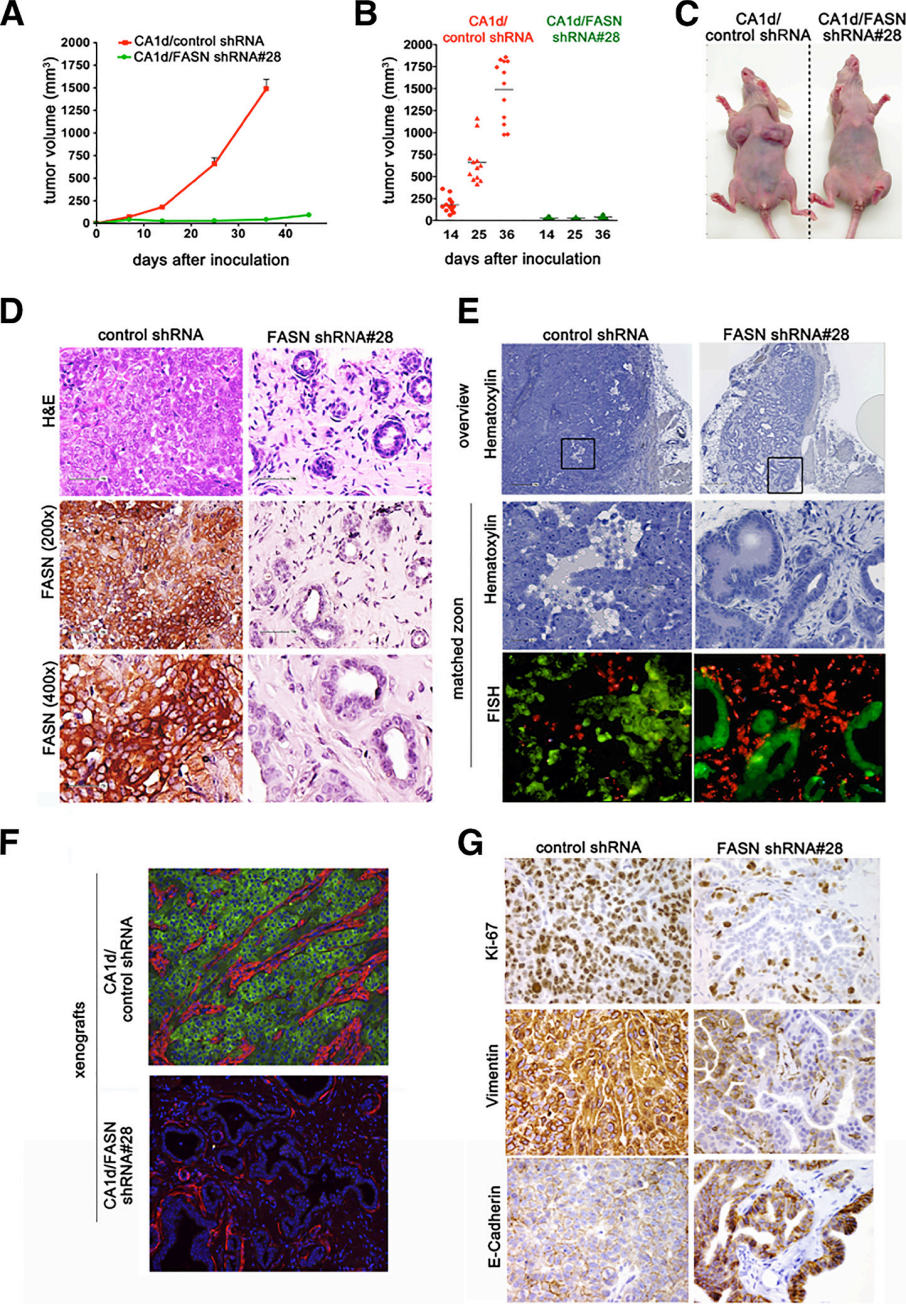
FASN knockdown in CA1d cells impairs tumor outgrowth and induces a dormant-like phenotype (**A**) FASN depletion inhibits tumor growth in an orthotopic model. Control (CA1d/vector control) and FASN-depleted cells (CA1d/FASN shRNA #28), injected into the fat pad of the mouse mammary gland, were monitored over the course of 7 weeks and tumor growth was assessed twice weekly. Tumor growth from one representative experiment out of three independent experiments, with similar results, is presented as mean ± SD. (**B**) FASN depletion decreases tumor volume. Distribution of tumor sizes from control and FASN-knockdown cells at 14, 25, and 36 days after injection. Note that only extremely small tumors were observed in FASN-depleted cells. Results are presented as mean ± SD (*n* = 12); *p* < 0.001. (**C**) Representative images of tumors originating from control and FASN-depleted cells in mice 6 weeks post injection. (**D**) Depletion of FASN restores a non-malignant, dormant-like, phenotype in CA1d cells. Histology of tumors derived from control (CA1d/vector control) and FASN-knockdown (CA1d/FASNshRNA#28) cells. Paraffin-embedded tumor sections were stained for H&E and FASN. Note the nearly normal looking ducts in the FASN-depleted cells. (**E**) Ducts from tumor-derived FASN-depleted cells are of human origin. Fluorescently labeled human (green) and mouse (red) home-brew whole genomic FISH probes were used to distinguish human and murine cells. (**F**) Visualization of invasive breast tumor cells by staining with smooth muscle actin (SMA, red) and fatty acid synthase (FASN, green). Note that in FASN-depleted tissues, a SMA-positive monolayer of cells surrounds a hollow lumen, while it is distributed within the tumor in control tissue. (**G**) FASN depletion reduces cell proliferation and reverses the aggressive tumor phenotype. Note high expression of vimentin, a marker for aggressive invasive tumors in control tumors, but not in tumors from FASN-depleted cells.

We next explored whether these findings had relevance to tumor formation *in vivo*. We injected tumor cells into the mammary fat pad of athymic nude mice and monitored tumor volume for several weeks (Figure 2A–2C; Figure S4A, S4B). CA1d control cells formed rapidly growing tumors, reaching volumes greater than 1500 mm^3^ 36 days after injection (Figure 2A–2C). In marked contrast, two independent FASN-depleted CA1d cell clones produced very small nodules with a volume of less than 100 mm^3^ in all injected sites 45 days after injection (Figure 2A–2C; Figure S4A, S4B).

Because the above data strongly suggested that normalization of the tumor cell phenotype *in vitro* was complemented *in vivo*, where the malignant potential is drastically reduced or completely lost, we performed a careful pathological examination of CA1d- and CA1D/FASN shRNA#28-derived tumors. Histopathological analyses revealed that FASN knockdown shifted the differentiation pattern of cancer tissues from an aggressive, mesenchymal-like phenotype in CA1d tumors to a highly differentiated, epithelial-like phenotype in FASN-depleted CA1d lesions (Figure 2D–2G). Whereas the high-grade tumors derived from FASN-positive CA1d control cells were large and pleomorphic with sheets of poorly differentiated cells forming nests, the lesions derived from FASN depleted-CA1d cells consistently displayed very small, low grade well-differentiated tissues resembling the normal glandular structure of the breast (Figure 2D).

The tissue architecture developing from CA1d- and CA1D/FASN shRNA#28 cells was re-evaluated by FISH to unambiguously prove that the ductal-like structures arising from suppression of FASN were of human origin. Human genomic FISH confirmed that FASN-overexpressing CA1d-derived tumors truly represented invasive human carcinomas, whereas the tumor tissues from FASN-depleted CA1d cells almost exclusively represented the normal duct-lobular system of the breast (Figure 2E). Assessment of the expression and localization of smooth muscle actin (SMA), a sensitive and specific myoepithelial marker commonly employed to facilitate the identification of basal-like tumors [35, 36], revealed a prominent distribution of SMA within the invasive carcinoma tissue of CA1d tumors (Figure 2F). FASN-depleted tissues, however, displayed SMA-positive myoepithelial cells surrounding a hollow lumen, a well recognized feature of normal ductal-lobular structures (Figure 2F). Consistent with the *in vitro* analysis (Figure S3), expression of the mesenchymal marker vimentin was suppressed in CA1D/FASN shRNA#28 cancer tissues, while E-cadherin levels were increased (Figure 2G), suggesting that loss of FASN imposes mesenchymal-like BC tissues to undergo the inverse plastic change that generates epithelial tissue, the so-called mesenchymal-epithelial transition (MET).

### Loss of FASN leads to a stable tumor reversion

**Figure 3:**
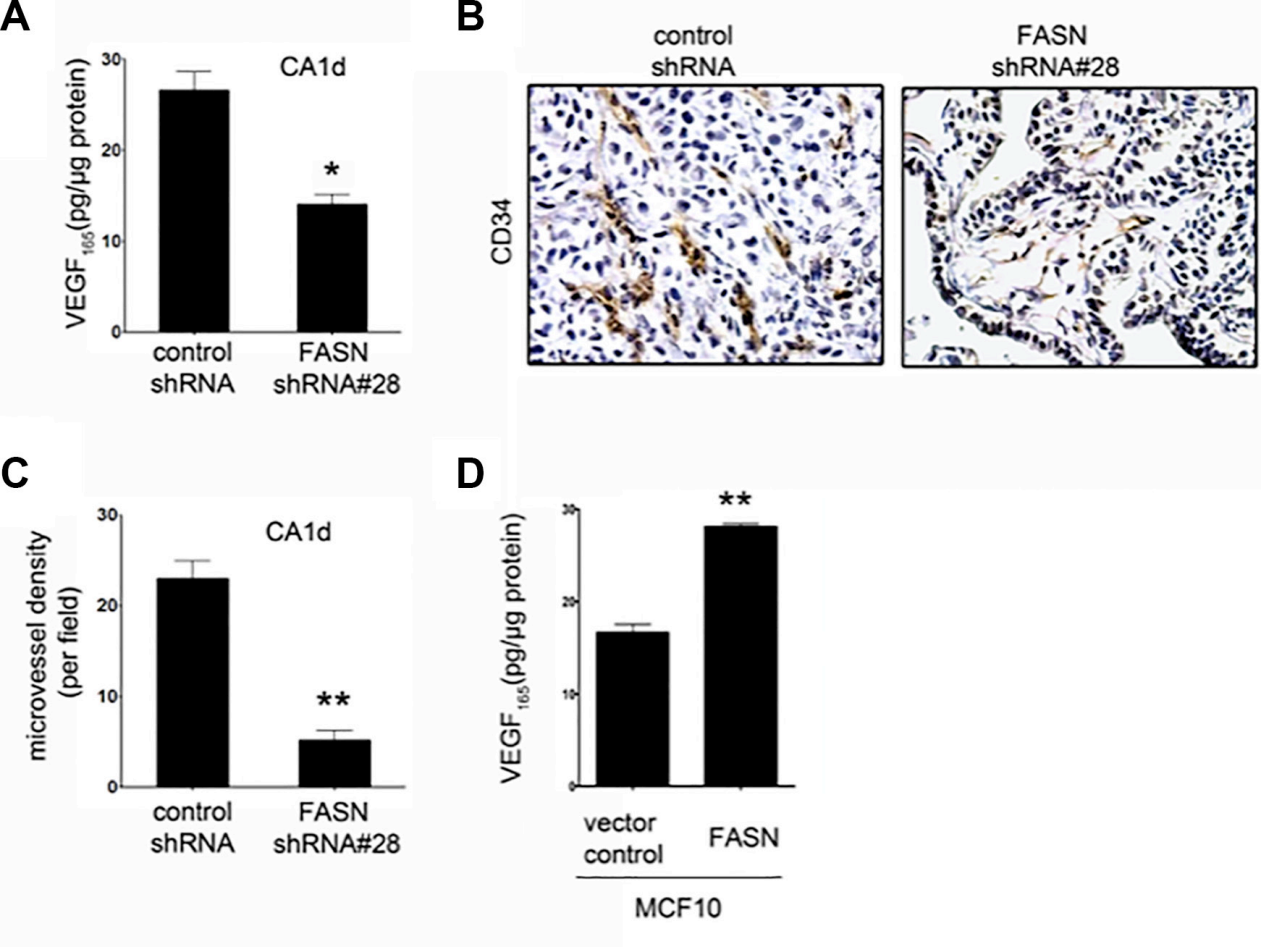
FASN depletion reduces VEGF secretion and angiogenesis in CA1d cells (**A**) ELISA-based analysis of VEGF in the supernatant from CA1d/vector control or FASN-deficient CA1d (CA1d/FASN shRNA#28) cells. Note that FASN knockdown results in a significant decrease in secreted VEGF. Data from one representative experiment are presented as mean ± SD; *p = 0.02. (**B**) Visualization of vascularization in tumors from control (vector) and FASN-depleted (FASN shRNA#28) xenografts by immunohistochemical staining for CD34. Note the reduced vasculature in FASN-deficient tumors compared with control tumors. (**C**) Quantification of one representative experiment as described in B. Results are presented as mean ± SD; **p < 0.001. (**D**) FASN overexpression increases VEGF secretion in non-transformed MCF10 cells. ELISA of VEGF in supernatant from MCF10 vector control or FASN over-expressing cells. Data from one representative experiment are presented as mean ± SD; **p < 0.001.

During the histopathological analysis, we observed that the percentage of Ki67-positive cells in the epithelial structures in FASN-depleted tissues was drastically lower than in FASN-overexpressing CA1d-derived invasive tumors (Figure 2G). These findings correlate well with the hypothesis that normal breast epithelial cells significantly reduce their proliferation rate after the formation of polarized alveoli and ducts, whereas primary BC cells and tumorigenic BC lines form large, disorganized and non-polarized colonies that fail to undergo growth arrest [32, 34, 37]. The recruitment of new blood supply is a rate-limiting step in tumor progression. Given that earlier studies in a 3-D model of breast carcinogenesis revealed that disorganized, proliferative transformed breast epithelial cells show significantly higher expression of angiogenic genes compared with their polarized, growth-arrested nonmalignant counterparts [38], we hypothesized that if FASN-dependent endogenous lipogenesis operates as a *bona fide* organizer of breast tissue architecture, FASN overexpression should activate the angiogenic switch, even in the absence of hypoxia. Conversely, FASN suppression should reduce the expression of angiogenic factors and the recruitment of endothelial cells (EC) to levels found in quiescent nonmalignant epithelium. Accordingly, FASN knockdown resulted in a significant down-regulation in the secretion of vascular endothelial growth factor (VEGF), a potent angiogenic factor, in CA1d cells (Figure 3A). Immunohistochemical assessment of CD34, an endothelial antigen commonly used to highlight the microvasculature vessel density (MVD) as a direct marker of the degree of neoangiogenesis, confirmed a markedly reduced capability of FASN-depleted tissues to recruit CD34^+^ EC compared with control CA1d-derived tumors (Figure 3B, 3C). In contrast, forced expression of FASN significantly increased VEGF production in MCF10A cells (Figure 3D).

**Figure 4:**
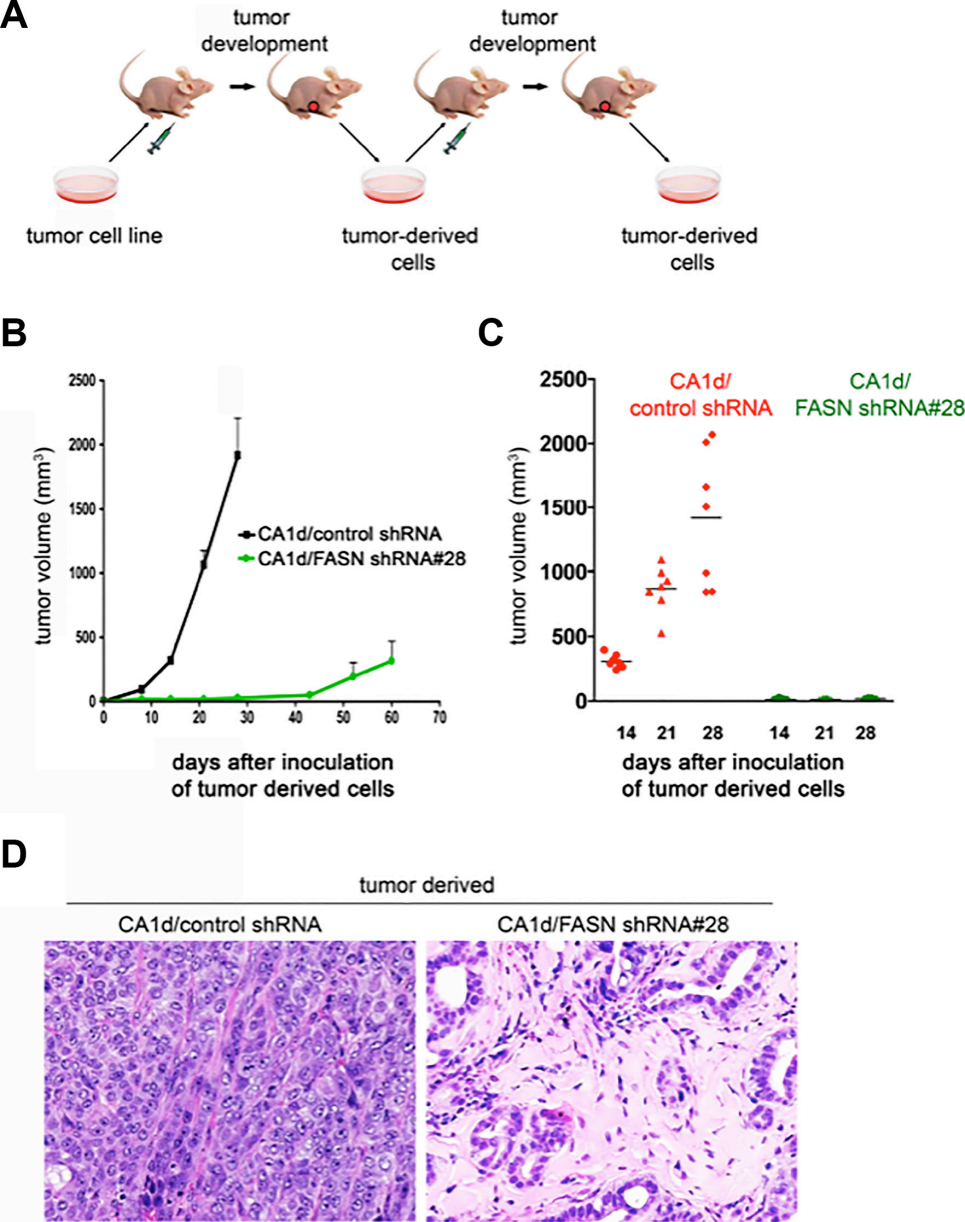
FASN depletion promotes a dormant-like phenotype as tumor-derived FASN-negative cells maintain a non-malignant phenotype after a second passage *in vivo* (**A**) Schematic representation of the experimental protocol to obtain second passage tumors. Control (CA1d/vector control) and FASN-depleted (CA1d/FASN shRNA #28) tumor-derived cells harvested from the first generation transplanted tumors after 7 weeks, were grown in culture, and xenografted to form second transplant-generation tumors. (**B**) Second generation, FASN-negative tumor-derived cells are growth impaired. Orthotropic model of tumor outgrowth of the second transplant generation of empty vector control (CA1d/vector control) and FASN-depleted CA1d tumor-derived cells (CA1d/FASN shRNA#28) measured over 60 days. Data from one representative experiment are presented as mean ± SD. (**C**) Graph showing the distribution of tumor sizes of second-generation tumors in control and FASN-depleted cells 14, 21, and 28 days post injection. Results from one representative experiment are presented as mean ± SD (*n* = 10). (**D**) Histology (H&E stain) of second passage tumors derived from control (CA1d/vector control) and FASN-negative (CA1d/FASN shRNA# 28) tumor-derived cells. Note that the FASN-negative cells maintain their non-malignant phenotype, presenting nearly normal looking ducts in contrast to the highly aggressive tumor in the control group.

Taken together, these findings are consistent with a mechanistic scenario in which, in the absence of FASN-catalyzed endogenous lipogenesis, breast epithelial cells might not retain sufficient cellular plasticity and will revert from an invasive phenotype to a dormant-like, quiescent state. To confirm that loss of FASN-driven exacerbated FA biogenesis irreversibly re-orientates tumor cells to a stable and permanent commitment to differentiation, we excised tumor tissues from CA1d and CA1D/FASN shRNA#28-injected animals (Figure 2) and serially transplanted them into mammary fat pats of athymic nude mice (Figure 4A). As anticipated, tumor-derived cells from CA1d control lesions generated fast growing tumors (Figure 4B, 4C), whereas tumor-derived cells from FASN-depleted lesions continued to form very small, slowly growing lesions that remained dormant for more than 30 days after injection (Figure 4B, 4B; Figure S4C, S4D). The histology of the tumors derived from transplanted cells was similar to that detected in the original tumors (Figure 4D). Consequently, CA1d control tumors displayed both highly invasive and proliferative phenotypes, while FASN-depleted tumors presented low-grade lesions with cells lining duct-like structures resembling the morphology of normal mammary gland (Figure 4D).

### FASN signaling regulates subcellular structure proteins that demarcate the epithelial or mesenchymal identify of breast epithelial cells

**Figure 5:**
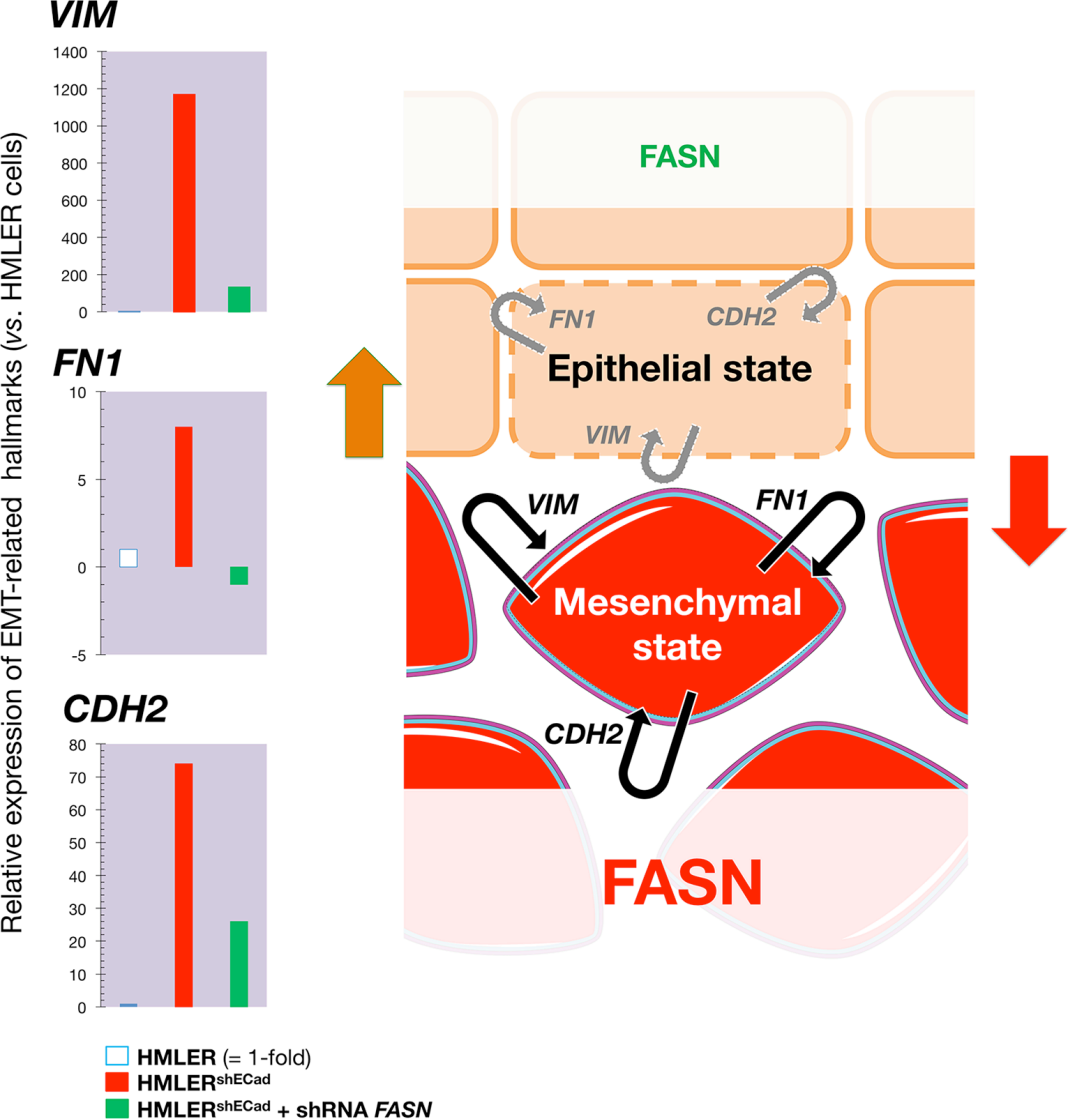
Transient knockdown of FASN gene expression suppresses structural hallmarks of EMT *Left*. Total RNA from HMLER, HMLER^shECad^, and HMLER^shEcCad^ + shRNA FASN cells was characterized in technical triplicates for the relative abundance of 19 mRNAs whose levels, in published results [76, 77], are significantly altered during activation/deactivation of the EMT genetic program. The transcript abundance of selected EMT-related genes (*VIM*, *FN1*, and CDH2) was calculated using the delta Ct method and presented as fold-change vs. basal expression in HMLER cells. *Right*. When breast epithelial cells acquire a fibroblast-like morphology during EMT, they not only down-regulate the obvious effectors of the epithelial phenotype such as E-cadherin (*CDH1*), but also switch the expression of an entire battery of genes including those that encode mesenchymal intermediate filaments (e.g., cytokeratins *à* vimentin switch) and ECM proteins (e.g, fibronectin, FN1). The status of FASN signaling appears to coincide with a self-reinforcing attractor (see Figure 7) in the structural configuration of the breast epithelial cell cytoskeleton, thus explaining that the sole correction of exacerbated lipogenesis can stably reprogram the malignant, invasive phenotype of cancer cells back to normal-like tissue architectures.

To explore the notion that FASN signaling might alter hallmark EMT effector molecules that determine the epithelial or mesenchymal identity of a breast epithelial cell, we took advantage of stable sibling cell lines in which an EMT has been induced to stably propagate CSC-like enriched populations. We used experimentally transformed HMLER breast cancer cells (human mammary epithelial cells [HMECs] overexpressing hTERT, SV40 T/t and H-RasV12) that had been modified to inhibit expression of the human *CDH1* (E-cadherin) gene *via* short hairpin RNA (shRNA; HMLER^shECad^ cells), which constitutes a valuable method for significantly enriching cells with CSC-like properties [39, 40]. Quantitative real-time PCR (qRT-PCR) analyses revealed that the stable repression of the epithelial hallmark *CDH1* was likewise accompanied by a dramatic up-regulation of vimentin (*VIM*) mRNA expression levels (> 1.000-fold) in HMLER^shECad^ cells, a gene coding for an intermediate filament component of the mesenchymal cell cytoskeleton (Figure 5). At the same time, the expression of genes coding for EMT-related subcellular structure proteins such as fibronectin (*FN1*) and N-cadherin (*CDH2*) were notably increased in *CDH1*-silenced HMLER^shECad^ cells.

A lentivirus-mediated small hairpin RNA-driven transient knockdown of *FASN* gene expression (3 days) was sufficient to notably reverse the overexpression of *VIM*, *FN1*, and *CDH2*. Remarkably, *FASN* inhibition almost fully reversed the exceptional high level of *VIM* expression (≈ 90% reduction) in EMT HMLER^shECad^ cells (Figure 5). The ability of FASN signaling to regulate hallmark EMT effector molecules was confirmed by the full suppression of the extracellular matrix (ECM) protein *FN1* and the significant downregulation (≈ 90% reduction) of the cell-surface protein *CDH2* in FASN-silenced HMLER^shECad^ cells. The ability of FASN inhibition to stably impose mesenchymal-like BC tissues to undergo the inverse plastic change that generates epithelial tissue *in vivo* (Figures 2–4), together with the fact that transient knockdown of FASN was sufficient to suppress hallmark structural and cytosolic/secretive proteins (vimentin, N-cadherin, fibronectin) in a model of EMT-induced CSC-like cells, strongly suggest that FASN signaling might play an unforeseen regulatory role in the structural configuration of the cytoskeleton and, hence, in determining the robustness of EMT *vs*. MET cell states (Figure 5).

### Pharmacological ablation of the FASN-driven lipogenic phenotype reverses malignant features of BC stem-like cells

Although the variety of FASN inhibitors developed in recent years (e.g., C75, C93, C246, FAS31, Orlistat, triclosan, GSK837149A) has consistently demonstrated preclinical activity in cultured cancer cell lines and xenograft models, none of these compounds have been tested in cancer patients because of limitations imparted by their pharmacological properties (e.g., poor cell permeability, poor oral bioavailability and lack of selectivity) or side-effect profiles (e.g., anorexia and weight-loss), which could be limiting in the development of cancer therapy [41–43]. Because our findings might be explained in terms of FASN-driven maintenance of an undifferentiated state in stem-like cells [44–46] within tumor populations, we evaluated whether therapeutically valuable drugs with a well-known FASN inhibiting activity that are currently available such as the anti-diabetic metformin [47–50] might significantly impact the malignant features of BC stem-like cells.

**Figure 6:**
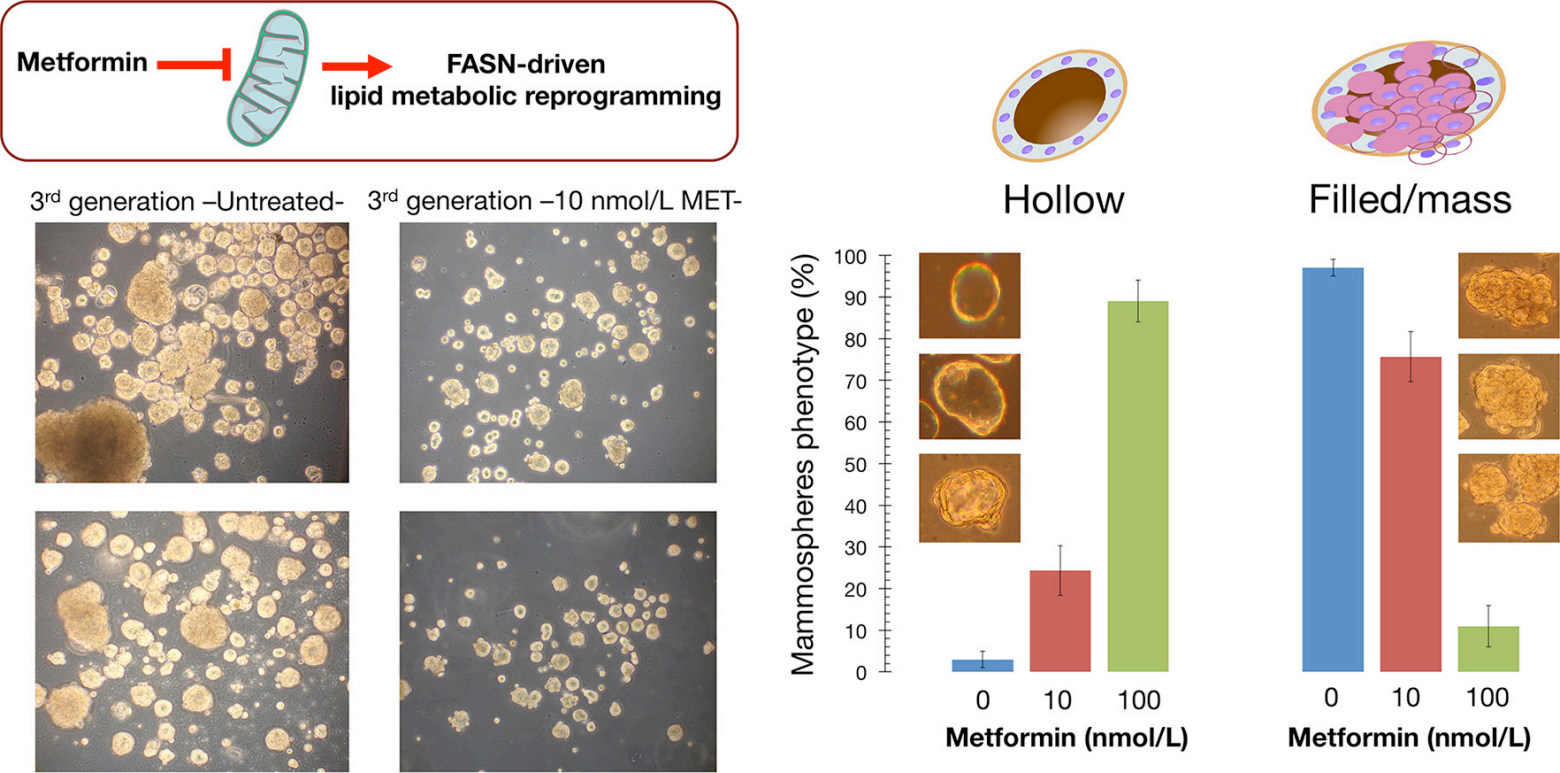
Indirect pharmacological inhibition of FASN promotes a phenotypic switch in breast tumorsphere architecture Left. Figure shows representative light microscope representations of 3rd generation mammospheres formed by MCF10DCIS.com cells growing in sphere medium in the absence or presence of graded concentrations of metformin, as specified (20× magnifications). *Right*. Quantification of the hollow (luminal-like) and filled/mass (basal-like) morphologies occurring in untreated and metformin-treated P3 mammospheres. Note that long-term exposure to clinically relevant nanomolar concentrations of the indirect FASN inhibitor metformin reduces the intra-sphere heterogeneity and promotes a switch from basal- to luminal-like sphere morphologies, suggesting promotion of a luminal differentiation axis and/or reversion of the basal/mesenchymal state.

We assessed whether metformin treatment altered the heterogeneity and morphology of tumorspheres derived from MCF10DCIS.com cells, a representative model of clinical comedo-ductal carcinoma *in situ* (comedo-DCIS) [51–53], a high-risk *in situ* breast lesion that might serve as precursor for basal-like BC. MCF10DCIS.com cells generated large, irregular solid spheroids that lacked a hollow lumen (Figure 6). To establish whether metformin was effective at altering the distinct basal-like mammosphere morphology comprising cohesive but loosely packed cells, MCF10DCIS.com were exposed to nanomolar concentrations of metformin over successive primary, secondary, and tertiary mammosphere generations. Interestingly, there was an increase in the number of smaller sized mammospheres relative to the larger ones over multiple generations of metformin-treated mammospheres. Moreover, the difference in the size of mammospheres apparently represented changes in cellular heterogeneity because a significant portion (up to 90%) of metformin-treated tertiary mammospheres became hollow spherical bodies composed of a single layer of epithelial cells surrounding an empty lumen (Figure 6). The drastically reduced intra-heterogeneity of tumorspheres occurring upon long-term exposure to clinically relevant concentrations of metformin, which mimicked the repeat dosing of drugs that patients would receive clinically, strongly suggest that indirect pharmacological inhibition of lipogenesis [47–50, 54] is sufficient to promote a switch from basal- to luminal-like sphere morphologies [55].

## DISCUSSION

We here show for the first time that FASN-catalyzed endogenous lipogenesis is a previously unrecognized organizer of breast tissue architecture wherein low levels of FASN activation furnishes a commitment to epithelial differentiation and increased FASN activation drives architectural destruction of breast tissue and progression to malignancy. Our results demonstrate not only that misregulation of lipogenic metabolism plays a causal role in progression of malignancy in human breast epithelial cells, but also that correction of exacerbated lipogenesis is sufficient to restore a normal-like tissue form and function and suppress tumorigenicity of metastatic BC cells *in vivo*. Thus, analogous to the epigenetic suppression of malignancy following retinoid treatment of leukemias, manipulation of endogenous lipogenesis can override the unstable cancer genome in metastatic tumor cells, offering a plausible alternative therapeutic modality of differentiation therapy for BC and other solid epithelial tumors.

**Figure 7:**
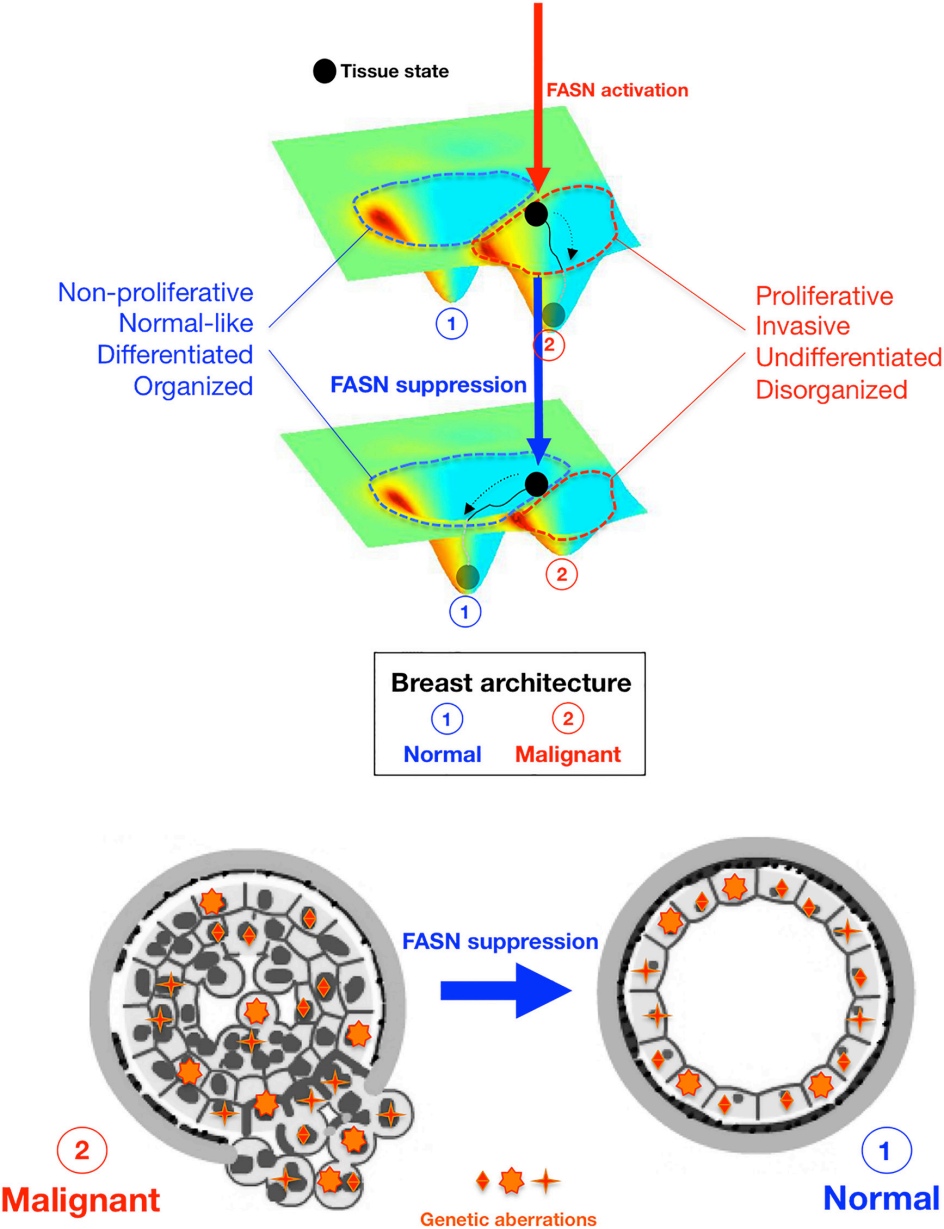
FASN-regulated phenotype of breast cancer tissues A Waddingtonian perspective. Schematic visualization of the principle that FASN-regulated network rewiring causes a distortion of the attractor landscape (i.e., tissue architecture) that results in shift of attractor boundaries and allows a non-genetic switching between discrete phenotypic states (i.e., N: Normal; M: Malignant). *Top*. Despite schematic reduction of dimensionality, the developmental trajectory on the Waddingtonian epigenetic surface is established by “tissue states”, which are defined not only by gene-gene network interactions at the cellular level but rather extends beyond to include changes in the cell-cell interaction network including neovascularization and alterations in ECM turnover and mechanics. The status of FASN activation (vertical arrows) causes a normal tissue to become proliferative and mesenchymal by placing the tissue state (black circle) within the metastatic basin of attraction (red, top panel). Upon FASN inhibition, the non-proliferative and epithelial attractor (blue, bottom panel) is enlarged at the cost of the metastatic attractor (red), which shrinks as a consequence of the suppression of FASN. *Bottom*. Suppression of FASN-driven endogenous lipogenesis is sufficient to toggle the metastatic phenotype back to the original state by acting as a controller of attractor switching between self-propelling and self-stabilizing tissue states irrespective of genetic alterations.

A survey of the genomic alterations in the isogenic MCF10 model of BC progression, from non-malignant MCF10A cells to pre-invasive DCIS.com cells and invasive CA1 cells, has recently revealed hundreds of coding mutations in multiple cancer driver genes commonly found in primary BC (e.g., PIK3CA and TP53) [56]. These mutations change the expression of genes involved in proliferation and adhesion, and in signaling pathways such as Wnt and MAPK, as well as the gain of copy-number alterations associated with cancer progression (e.g., deletion of RUNX1, a lineage specific master regulator controlling mammary luminal cell fate). In this setting, our discovery of FASN as a negative regulator of tissue architecture and terminal epithelial cell differentiation, which is dominant over the malignant phenotype of metastatic tumor cells possessing multiple cancer-driving genetic lesions [28, 56], points to an unappreciated role for endogenous lipogenesis in controlling a supra-genetic dimension critical to tumorigenesis. That the correction of a metabolic cue, such as endogenous lipogenesis, can reverse the malignant behavior of mesenchymal-like metastatic BC cancer tissues to recover normal tissue architecture in a genome background that is malignant and unstable due to *bona fide* cancer-driving genetic abnormalities, obliges a consideration of a conceptual Waddingtonian framework of system-level dynamics based on a “state space” model of self-organizing “attractors” (i.e., phenotypes), instead of the mainstream genetic determinism that underlies the most commonly claimed models of metastatic progression [57–59]. Furthermore, the ability of FASN to dictate the organizational phenotype of breast tissue and the fate of normal and neoplastic epithelial cells might not be satisfactorily explained exclusively in terms of reprogrammed phenotypes (i.e., mesenchymal *versus* epithelial) or behaviors (metastatic *versus* non-invasive) at the cellular level. That is, solely a model of “attractor switching” applied to interactions at the tissue level [60] can clarify how the suppression of lipogenic signals is sufficient to guide genetically-aberrant metastatic tumor tissues to a near normal phenotype in terms of organized and polarized epithelial structures with low proliferation index and angiogenic *shut off*. Even subtle changes in FASN-driven endogenous lipogenesis may translate to a change in the attractor landscape with major biological consequences if the induced network rewiring results in shifts of attractor boundaries and shapes (Figure 7). Correction of exacerbated lipogenesis can cause a distortion of the attractor landscape that easily allows cells in a given tissue organization attractor that encode a proliferative, mesenchymal-like undifferentiated phenotype, to suddenly acquire a new phenotype by placing them in a self-stabilizing attractor encoding a quiescent, mature epithelial-like tissue phenotype. Conversely, upon activation of FASN-driven endogenous lipogenesis, an *a priori* inaccessible attractor encoding the mesenchymal-like undifferentiated tissue phenotype can suddenly become easily accessible to cells originally placed in the self-stabilizing attractor of terminal tissue differentiation. Because cellular plasticity is at the center of phenotypic reversion of the malignant phenotype, which is also linked to the concept of tumor cell stemness, it is formally possible that the activation status of FASN might dictate the degree of refractoriness of breast epithelial cells to differentiation and, therefore, their intrinsic susceptibility to the epigenetic rewiring required for the activation of a pathological differentiation program of aberrant stemness.

Mutational and epigenetic changes in oncogenes and tumor suppressor genes have been investigated exhaustively during the past decades as the main molecular players involved in the maintenance of tissue homeostasis and, consequently, of normal *versus* cancerous organization and/or non-metastatic versus metastatic behavior. Nonetheless, largely unknown extracellular and/or intracellular cues not necessarily dependent on (epi)genetic alterations can drastically impact the intrinsic developmental uncertainty of tissues, promoting cell fate switching by an asymmetric amplification of certain processes and events to be initiated at the correct site and appropriate time (e.g., normal tissue morphogenesis and functional differentiation), in conjunction with long-range inhibition of pathological developmental programs (e.g., dedifferentiation and loss of tissue architecture). The pathways that regulate cell architecture and tissue organization are, unfortunately, poorly understood and have been largely disregarded by biomedical scientists and clinicians as a means to identify molecular targets for long-term management of human carcinomas. Moreover, the majority of studies have focused on the role of signaling emanating from the extracellular matrix (ECM) as the ultimate regulator of tissue architecture and, hence, of epithelial function. In this regard, landmark 3D culture modeling approaches from the Bissell group have elegantly demonstrated the feasibility of phenotypically altering the ECM to dramatically modify the differentiation state of invasive tumor cells to one resembling a more normal cell phenotype irrespective of the genetic abnormalities in the tumor cells [61–67]. Here, we have mimicked the ability of cell surface ECM-receptor manipulations to abrogate malignancy and revert the tumor phenotype to a near-normal phenotype by suppressing cancer-associated overactive endogenous lipogenesis [68, 69]. Our findings confirm the notion that several canonical signaling pathways are indeed intrinsically and reciprocally linked within the morphological normal form, irrespective of the strategy used. Our results corroborate recent studies showing that blocking FASN-driven lipid synthesis efficiently overcomes tumor regrowth and metastasis after antiangiogenic therapy withdrawal [70]. We cannot exclude the possibility that the ability of FASN to regulate tumor vasculature through alteration not only of the profile of secreted angiogenic factors, but also of expression and activity of key regulators of tissue architecture such as matrix metalloproteinases (e.g., MMP-9) [71], might establish a dynamic reciprocity between FASN-driven endogenous lipogenesis, tissue polarity (e.g., changes in intermediate filaments such as the EMT-related cytokeratin-to-vimentin switch), ECM integrity, and angiogenesis, thus providing an axis for either tissue homeostasis or malignant progression [72–74]. Although it might appear counterintuitive, the “easy” way by which tissues switch between the mesenchymal and epithelial phenotypes in response to changes in the activation status of FASN might reveal that, in Waddingtonian terms, an “attractor” in the biochemical gene activity profile (e.g., FASN-regulated effectors of epithelial phenotype) is coincidental with a self-reinforcing “attractor” in the structural configuration of the cytoskeleton, thus explaining the robustness of phenotypic reversion and normalized differentiation that occurs in response to FASN suppression in metastatic cancer cells.

Our current findings establish FASN as a critical link between cell metabolism and the control of tissue architecture, the elucidation of which could have an important impact on targeted therapy for malignancies of the breast. Indeed, the fact that sole correction of FASN upregulation, one of the most common and earliest metabolic changes in human malignancies that is not caused by mutational or gene amplification events, can reprogram metastatic cancer cells to stably recover normal-like tissue architecture, might open a new avenue to chronically restrain the life-threatening potential of metastatic carcinomas by using FASN-based differentiation therapies.

## MATERIALS AND METHODS

### Cell lines, cell culture and reagents

MCF10A, MCF10NeoT, MCF10AT1 and MCF10A1kcl2 cells were cultured in DMEM/Ham's F-12 (DMEM/F-12) supplemented with 5% horse serum (HS), 10 mM HEPES, 10 ng/ml insulin, 20 ng/ml EGF, 100 ng/ml cholera toxin, and 0.5 mg/ml hydrocortisone. MCF10ADCIS, MCF10CA1a (CA1a), MCF10CA1b (CA1b), and MCF10CA1d (CA1d) cells were cultured in DMEM/F-12 with 5% HS. Cells were maintained at 37°C in a humidified atmosphere of 95% air and 5% CO_2_. Cells were authenticated to ensure their identity using the short tandem repeat profiling method provided by the Genotyping Shared Resource, Mayo Clinic (Rochester, MN). Cells were regularly tested to confirm the absence of mycoplasma using the MycoAlert^™^ mycoplasma detection kit (Lonza, Walkersville, MD).

### Generation of stable cell lines

MCF10CA1a and MCF10CA1d cells (12 000/well) were seeded in 24-well plates in DMEM containing 10% fetal bovine serum. After 24 h, cells were washed with PBS and the medium was changed to DMEM/F12 containing 5% HS and 8 μg/ml polybrene. Cells were then infected with the lentiviral pLKO.1 vector (vector control) or pLKO.1 vector carrying shRNA targeting FASN at MOI = 5 (TRCN0000003126, TRCN0000003127, TRCN0000003128 and TRCN0000003129) or with lentiviral transduction particles expressing a control scramble shRNA (MISSION, Sigma, Burlingame, CA). After transduction, the medium containing the lentivirus was replaced with fresh medium containing puromycin (0.75 μg/ml) as a selection marker.

### Western blotting

Equal amounts of protein (10 μg) were resolved on 10% Criterion XT Bis-Tris Precast Gels (Bio-Rad, Hercules, CA) and transferred to PVDF membranes. Membranes were blocked with TBS-T (TBS and 0.5% Tween 20) containing 5% (w/v) non-fat dry milk or 5% BSA (depending on the primary antibody) for 30 minutes to 1 h and washed with TBS-T. Membranes were then incubated overnight at 4°C with different antibodies including FASN (clone 23, BD Biosciences, Pharmingen), p21 (F-5, Santa Cruz Biotechnologies, Santa Cruz, CA) and AMPK, p-AMPK, ACC and p-ACC (Cell Signaling Technology, Danvers, MA). After a further 3 washes with TBS-T, membranes were incubated with horseradish peroxidase-linked secondary antibodies. Blots were then stripped and re-probed with a monoclonal antibody to β-actin and horseradish peroxidase-linked goat anti-mouse IgG secondary antibody. Proteins were detected by an enhanced chemoluminescence reaction using Hyperfilm (Amersham-Pharmacia, Piscataway, NJ).

### Cell viability assay

Cell viability was determined with the MTT (3-(4, 5-dimethylthiazol-2-yl)-2,5-diphenyl tetrazolium bromide) reduction assay. Briefly, cells (4 × 10^3^/well) were seeded into 96-well plates and allowed to attach for 16 h. After a PBS wash, fresh medium containing cerulenin or C75 in various concentrations was added. Three days after treatment, the cells were washed, supplied with fresh medium containing 15 μl of MTT dye and then incubated for 4 h at 37°C. The reaction was stopped by addition of 100 μl solubilizing solution/stop mix and the plates were incubated overnight at 37°C. The following day, the absorbance at 570 nm was measured in each sample using a multiwell plate reader (Victor ×3, PerkinElmer) and the cell viability was assessed using the equation: (A_570_ of Cerulenin or C75 treated sample/A_570_ of vehicle-treated sample) × 100.

### *In vitro* growth assays

Anchorage-dependent growth assay: Cells (5 × 10^3^/well) were seeded into 24-well plates (Cellstar, Greiner Bio-One) in DMEM/F-12 supplemented with 5% HS. After 24 hours, cells were counted (= day 0) and counting was continued each day for 4 days using the Vi-cell XR cell counter and viability analyzer (Beckman Coulter, CA). Anchorage-independent growth assay: A bottom layer of 1.5 ml DMEM/F-12 medium containing 0.6% agar and 5% HS was prepared in 6-well plates. After the bottom layer solidified, cells were suspended in a 1 ml top layer of DMEM/F-12 medium containing 0.35% agar and 5% HS. Plates were then incubated in a humidified 5% CO_2_ incubator at 37°C. After 9–14 days, colonies larger than 50–70 μm in diameter were counted with an automatic colony counter (Gel Count, Oxford Optronix, UK).

### Three-dimensional (3D) basement membrane assay

Cells were cultured using a 3D overlay method as described [75]. Briefly, cells were seeded (3000 cells/well) on a solidified layer of growth factor-reduced Matrigel (BD Bioscience) measuring approximately 1–2 mm in thickness in an 8-well chamber slide. The cells were overlaid with DMEM/F12 medium containing 5% HS and 2% Matrigel and grown for 6 days.

### Indirect immunofluorescence

Staining was performed as described for MCF10A acini cultured in Matrigel [75] using anti E-cadherin (BD Transduction Laboratories) as primary antibody and Alexa-488 conjugated anti-mouse from Molecular Probes as secondary antibody. Nuclei were visualized using DAPI.

### Animal studies

Two million cells (cell type as indicated in the description for each experiment) in 1:1 PBS/Matrigel (10 mg/ml, BD Bioscience) were injected into the mammary fat pad of 3–4-week-old female athymic nude-Foxn1^nu^ mice (Harlan, Indianapolis, IN). Tumor volume was calculated by 3D measurements using the formula:

$$\text{Tumor volume}({\text{mm}}^{3})=\frac{\text{length}\times \text{width}\times \text{height}}{2}$$

Tumor measurements were performed weekly using a Vernier caliper. Mice were sacrificed before the tumor volume reached ~ 2 cm^3^. Mice were then euthanized, tumors were excised, fixed in formalin, and embedded in paraffin. Paraffin-embedded tumor sections were then prepared and stained with hematoxylin and eosin for histopathology analysis. Tumor measurement was blinded to minimize experimental bias. Additional sections were generated for immunohistochemical analysis. The Mayo Institutional Animal Care and Use Committee approved the animal protocols.

### Preparation of tumor-derived cells

CA1d/vector control or CA1D/FASN siRNA tumors grown in the mammary fat pad of athymic nude mice for 7 weeks were cut in small pieces, incubated in a trypsin solution at 37^°^C for 10 minutes and resuspended in 10 ml of culture medium. Cells were collected by centrifugation at 400 g for 5 minutes and plated on a collagen-coated dish. Cells were cultured in DMEM/F-12 with 5% HS, penicillin (100 units/ml), streptomycin (100 μg/ml), puromycin (1 μg/ml) and amphotericin B (2.5 μg/ml) at 37^°^C in a humidified 5% CO_2_ atmosphere until they reached 70% confluence, after which they were re-plated in Petri dishes without collagen. After two weeks of culture, the expression of FASN in tumor-derived cells was evaluated by western blotting.

### VEGF production measured by ELISA

Cells (2 × 10^5^/well) were seeded in 6-well plates and cultured in complete growth medium. After reaching ~70% confluence, cells were washed twice with PBS and the medium was changed to serum-free medium (SFM). After 24 hours of serum starvation, culture media was collected, centrifuged at 1100 g for 10 minutes at 4^°^C and the supernatant was tested for VEGF immediately or stored at −80°C until analysis. VEGF protein was determined using the VEGF ELISA Kit (Peprotech, Rocky Hill, NJ). Absorbance was measured at 405 nm in an ELISA reader (Bio-Tek Instruments, Winooski, VT).

### Immunohistochemical analysis

Paraffin sections were deparaffinized at 60°C for 60 minutes. For Ki-67 and E-cadherin detection, slides were subjected to heat-induced epitope retrieval (HIER) in a decloaking chamber (Biocare Medical, Concord, CA) with Target Retrieval solution, pH 6.0 (DakoCytomation, Glostrup, Denmark). Antigen retrieval for FASN was performed with 10 mM sodium citrate, pH 6.0 for 20 minutes at 95^°^C and citrate-EDTA buffer (10 mM Citric Acid, 2 mM EDTA, pH 8 was used for CD34. Endogenous peroxidase activity was blocked with 3% hydrogen peroxide for 5 min. Non-specific binding sites were blocked using PBS-T (PBS 0.01 M, triton ×−100 0.1%, Tween 20 0.05%) containing 1% BSA and 5% HS. Slides were exposed for 1.5 hours at room temperature to either rabbit polyclonal anti-Ki-67 (Fisher) or rabbit polyclonal anti-E-Cadherin (Cell Signaling). Sections were incubated at 4^°^C overnight with anti-FASN Rabbit IgG1 (IBL) or anti-mouse CD34 Rat IgG1 (BD Pharmingen). Thereafter, the slides were rinsed three times with PBS and incubated with the corresponding secondary antibodies (anti-rabbit or anti-mouse biotin-labeled; DakoCytomation) followed by streptavidin–biotin–peroxidase for 30 minutes at room temperature. Immunostaining was visualized using the DAB+ Kit (DakoCytomation). Slides were counterstained using Gill's haematoxylin. Microvessels were identified by immunostaining with rat anti-mouse polyclonal antibody CD34. The entire section was scanned systematically at low magnification (×100) to identify the most intense areas of neovascularization (“hotspots”) within the tumor. After five hotspots areas with the highest number of capillaries and small venules were identified, microvessels were counted at high power magnification (×400) and the average count in five fields was calculated.

### Immunofluorescence analysis

Paraffin sections were deparaffinized at 60°C for 60 minutes; antigen retrieval and blocking was performed as described for the immunohistochemical analysis. Anti-FASN antibody was used as described above and the anti-SMA mouse monoclonal antibody (Dako) was diluted in PBS containing 3% non-fat dry milk. After three washes in PBS, the signal was detected by a combination of the anti-rabbit FITC (green) and the anti-mouse TRITC antibodies (red, Sigma).

### Quantitative real-time polymerase chain reaction (qRT-PCR)

The total RNA was extracted from cell cultures using a Qiagen RNeasy kit and QIAshredder columns according to the manufacturer's instructions. One microgram of total RNA was reverse-transcribed to cDNA using the Reaction Ready^™^ First Strand cDNA Synthesis Kit (SABiosciences) and applied to a customized PCR array (96-well format) containing the following panel of genes: *GSC, KRT14, KRT19, NUMB, TCF3, TCF4, SDC1, ZO-1, CD44, TWIST, SNAI1, VIM, SLUG, CDH1, ZEB1, CDH2, ZEB2, FN1*, and *CD24*. The arrays were processed according to the SABiosciences RT-PCR manual and analyzed using an Applied Biosystems 7500 Fast Real-Time PCR System with an automated baseline and threshold cycle detection. The data were interpreted using SABiosciences's web-based PCR array analysis tool.

### Lentiviral transduction

Pre-packaged lentiviral particles that either encoded a non-targeting shRNA (negative shRNA, sc-108080) or sequences specifically targeting the human *FASN* gene (sc-43758-V; Lot#B2610) were purchased from a commercial provider (Santa Cruz Biotechnology). For viral infection of HMLER^shECad^ cells, the regular medium was replaced with culture medium containing 5 μg/mL polybrene (Santa Cruz Biotechnology, sc-124220). Cells were then exposed to lentiviruses for 48 h. The cells were then washed and grown in regular médium for an additional 24 h.

### Mammosphere culture and mammosphere-forming efficiency

Single cell suspensions (1000 cells/mL) were plated in 6-well tissue culture plates previously coated with poly-2-hydroxyethyl-methacrylate (Sigma, St. Louis, MO) to prevent cell attachment, in serum-free DMEM/F-12 supplemented with 1% L-glutamine, 1% penicillin/streptomycin, 2% B27 (Invitrogen, Carlsbad, CA), 20 ng/mL EGF (Sigma) and 20 ng/mL FGFb (Invitrogen). The medium was made semi-solid with 0.5% methylcellulose (R&D Systems, Minneapolis, MN) to prevent cell aggregation.

Nonadherent spherical clusters of cells, named P1, were collected after 7 days and disaggregated using enzymatic and mechanical dissociation. P1-derived single-cell suspensions were seeded again at 1000 cells/mL to generate nonadherent spherical clusters of cells, named P2, and the process was repeated at third time to generate P3. This procedure was performed in the absence or presence of graded concentrations of metformin (0, 10, and 100 nmol/L). Mammosphere-forming efficiency (MSFE) was calculated as the number of sphere-like structures (diameter > 50 μm) formed by P1, P2, and P3 cells divided by the original number of cells seeded, and expressed as a percentage.

### Statistical analyses

The quantitative data were collected from at least three independent experiments performed in triplicate and the data from one representative experiment are presented as mean ± S.D. For comparisons between two populations, an unpaired two-tailed Student's *t* test was performed from three independent experiments, unless otherwise specified. Statistical analysis was performed with GraphPad Prism 5.01 or Microsoft Office Excel software. Differences were considered significant when the *p* values were < 0.05.

## SUPPLEMENTARY MATERIALS FIGURES


