# Supplementary material for: Suppression of endogenous lipogenesis induces reversion of the malignant phenotype and normalized differentiation in breast cancer
Source: Oncotarget. 2016 May 18;7(44):71151–68. doi: 10.18632/oncotarget.9463 (PMC5342069; doi:10.18632/oncotarget.9463)
Supplement: Supplementary file 1 [file oncotarget-07-71151-s001.pdf]

Suppression of endogenous lipogenesis induces reversion of the malignant phenotype and normalized differentiation in breast cancer

Supplementary Materials

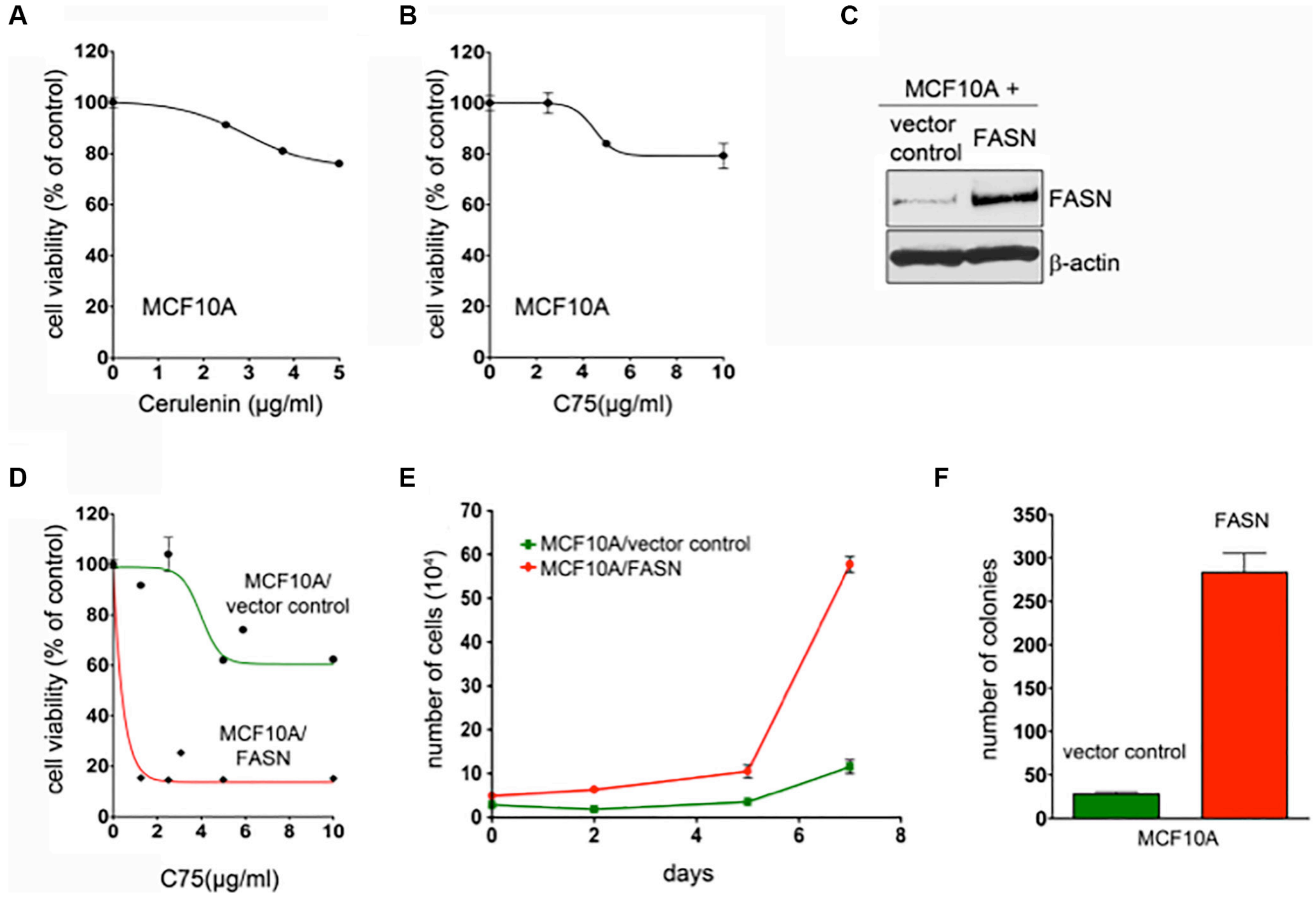

Supplementary Figure S1: Overexpression of FASN in MCF10A cells confers a more aggressive phenotype by promoting a proliferative advantage. (**A**, **B**) FASN-negative MCF10A cells are insensitive to FASN inhibitors. Cells were treated with cerulenin (A) or C75 (B) for 24 h as indicated and cell viability was assessed by MTT reduction. Data from one representative experiment is presented as mean ± SD. (**C**) Representative western blot confirming overexpression of FASN in MCFA/FASN cells compared with vector control cells. (**D**) FASN overexpression sensitizes cells to anti-FASN drugs. MCF10A control (vector control) and MCF10A/FASN cells were treated with cerulenin as indicated and cell viability was assessed by MTT reduction. Data from one representative experiment is presented as mean ± SD; $p = 0.02$ (day 10). (**E**) FASN overexpression results in a growth advantage over control cells. Cell growth in vector control and FASN overexpressing cells was monitored over 7 days and the number of cells was assessed by trypan blue exclusion. Data from one representative experiment is presented as mean ± SD; $p < 0.009$. (**F**) Overexpression of FASN increases anchorage-independent growth, supporting a more aggressive phenotype. MCF10A/vector control and MCF10A/FASN cells were plated in soft agar for three weeks and the number of colonies was determined using a colony counter. Data from one representative experiment is presented as mean ± SD; $p = 0.04$.

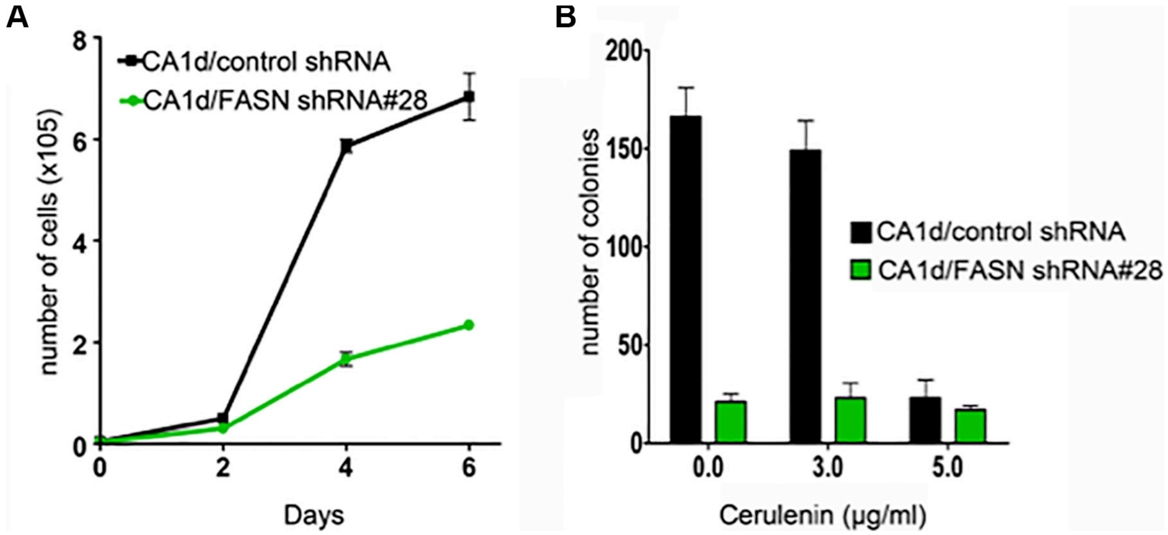

Supplementary Figure S2: Characterization of FASN-depleted cells. (**A**, **B**) FASN depletion reduces cell viability and proliferation. A. Control (CA1d/vector control) and FASN-negative (CA1d/FASN shRNA#28) cells were monitored over 6 days and the number of cells was assessed by MTT reduction. Data is presented as mean ± SD from one representative experiment. B. CA1d/vector control and CA1d/FASN shRNA#28 cells were treated with cerulenin as indicated and cell growth was determined with a soft agar assay as described in Figure S1. Two independent experiments were performed and the results from one representative experiment are presented as mean ± SD.

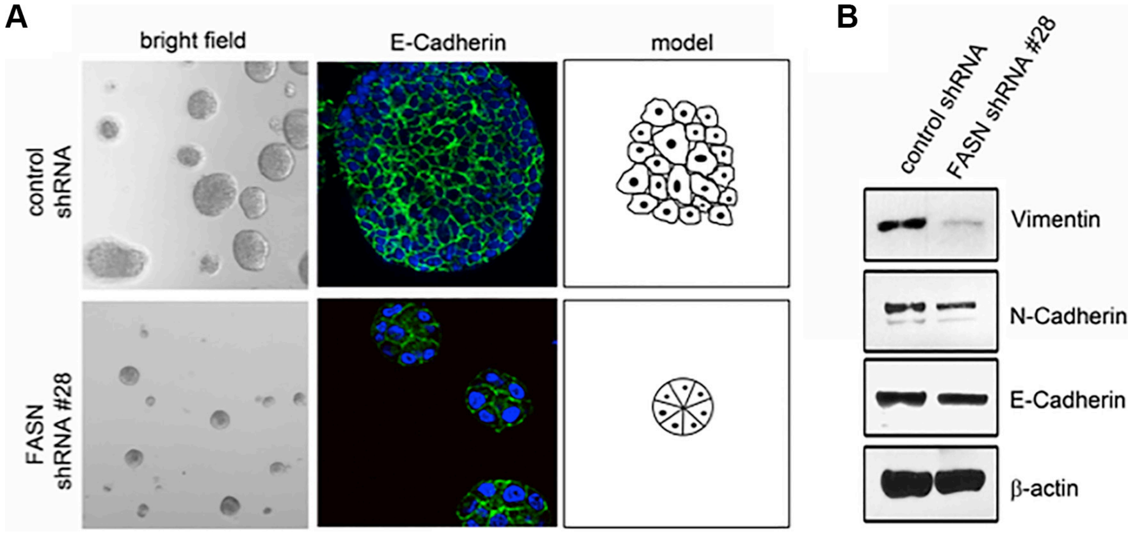

Supplementary Figure S3: FASN knockdown attenuates cell plasticity of CA1d cells in 3D Matrigel cultures. (**A**) *Left panel*. Phase contrast images of control (vector control) and FASN- depleted cells (FASN shRNA#28) after 7 days of culture in a 3D Matrigel model. *Middle panel*: E-cadherin localization in each colony/sphere was analyzed by confocal microscopy. Note that the control cells appear disorganized while the FASN-depleted cells show a more duct-like morphology. Nuclei were visualized with DAPI (blue). *Right panel*: Model showing the different phenotypes of invasive breast cancer cells and normal cells grown in 3D culture. (**B**) Knockdown of FASN substantially downregulates the mesenchymal marker vimentin. Western blot showing the expression of E-cadherin, vimentin and N-cadherin in control (CA1d/vector control) and FASN-knockdown (CA1d/FASN shRNA#28) cells after 7 days in Matrigel.

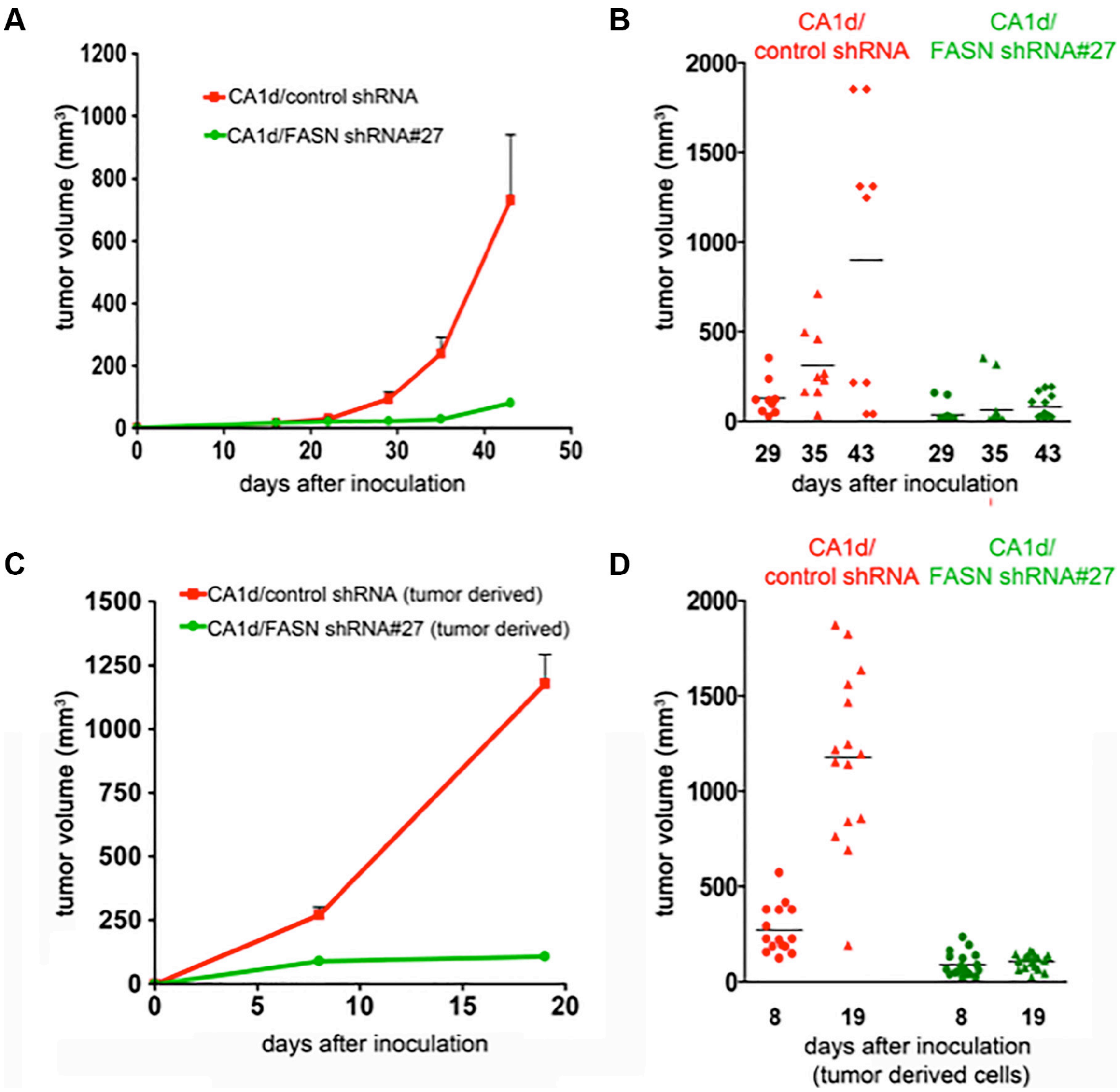

Supplementary Figure S4: FASN knockdown inhibits tumor growth even in second passage-derived tumor cells. (**A**) Orthotropic tumor outgrowth of control (CA1d/vector control) and FASN-depleted CA1d cells (CA1d/ FASN shRNA # 27) measured over 43 days. Tumor growth was monitored twice weekly and was significantly attenuated in FASN-deficient cells. (**B**) Distribution of tumor sizes from control and FASN-depleted cells at day 29, 35, and 43 after injection. Results are presented as mean ± SD; $p < 0.001$. Note that tumor volume is significantly lower in the FASN-negative cells compared with control cells. (**C**) FASN depletion and inhibition of tumor growth is maintained in second passage tumor-derived cells. Control (CA1d/vector control) and FASN-knockdown (CA1d/shRNA FASN #27) tumor-derived cells were collected from first transplant-generation tumors 45 days post transplantation, grown in culture and subsequently xenografted to form second transplant generation tumors. Tumor growth was monitored over 20 days and is significantly inhibited in the tumor-derived FASN cells compared with tumors originating from control cells. (**D**) Distribution of tumor sizes from second passage tumors at 8 and 19 days post-injection. Results are presented as mean ± SD. Note that FASN-depleted cells consistently have a lower tumor burden than control cells.
